# Polymer Clay Nano-composites

**DOI:** 10.3390/polym11091445

**Published:** 2019-09-03

**Authors:** Stefano Leporatti

**Affiliations:** CNR Nanotec-Istituto di Nanotecnologia c\o Campus Ekotecne via Monteroni, 73100 Lecce, Italy; stefano.leporatti@nanotec.cnr.it

Clay–polymer composite materials is an exciting area of research and this Special Issue aims to address the current state-of-the-art of “Polymer Clay Nano-Composites” for several applications, among them antibacterial, environmental, water remediation, dental, drug delivery and others. The original scope of the Special Issue was comprehensively devoted to the synthesis and characterization of polymer clay nano-composites employed for several applications, including nano-clay polymer composites and hybrid nano-assemblies. Furthermore, polymers can be loaded with clay nanoparticles creating novel composite nano-materials enhancing composite strength features. The issue is composed of 16 contributions, fifteen articles and one review. They can be conveniently divided into one group related to *Halloysite-composites* (four papers including one review), a second group which deals with *Montmorillonite-composites* (four papers) and a third group, which can be generically referred to *Hybrid Clay Nano-Composites* (eight contributions). 

Relative to the group of Halloysite-composites, in the review [1] Wu et al. summarized the recent progress toward the development of polysaccharide-HNTs composites, paying attention to the main existence forms and wastewater treatment application particularly. The purification of Halloysite Nanotubes (HNTs) and fabrication of the composites were also discussed. Furthermore, they reported the unique characteristics of polysaccharide-HNTs composites and reviewed the recent development of the practical applications. In particular they pointed out that (1) polysaccharide-HNTs composites have improved mechanical, thermal, and swelling properties and good biocompatibility. Therefore they are promising nano-fillers for high-performance polymer composites. (2) HNTs can be combined with polysaccharides by different methods (3) the degree of dispersion of HNTs and the interfacial interactions between polysaccharides and HNTs are key factors affecting the performance of composites. (4) Polysaccharide-HNTs composites has shown promising potential for biomedical applications. Another contribution from Xiandong Zhang and Guangshun Wu [2] dealt with HNTs Carbon Fiber (CFs) composites. The authors achieved for the first time the chemical grafting of halloysite nanotubes (HNTs) with amino or carboxyl groups onto the CFs surface, which was aimed to enhance the composites interfacial strength. Functional groups of HNTs and fiber surface structures were characterized, as well as interfacial properties and anti-hydrothermal aging behaviors. Interfacial reinforcement mechanisms for untreated and modified CF composites were also compared and discussed. Morphology, mechanical properties, water resistance and optical properties of the Fish gelatin (FG)/glycerol (GE)/halloysite (HT) composite films were investigated by Qiang et al. in [3]. Interestingly, they showed that with increasing GE content, the elongation at composite breaks increased significantly, but their tensile strength (TS) and water resistance decreased. Their results indicated that the addition of GE greatly improved film flexibility with a decrease in the TS of the film. Moisture uptake and water solubility were also improved by the addition of GE into the FG matrix, indicating that the water-resistance of the film decreased due to the GE added. Furthermore, the presence of GE enhanced the dispersion of HTs in the FG matrix and thus enhanced the properties of the obtained composite films. In the last paper of this HNTs-composites group, Lvov et al. [4] wanted to answer the following question: What additives could be used to increase the strength of silica gels? To answer this, they prepared colloidal silica gels with various additives and they measured gel strength. It was found that cellulose nanofibrils considerably increased the gel strength. Furthermore, cellulose nanofibrils could be produced from cheap industrial-grade cellulose with low-cost industrial chemicals. Therefore, cellulose nanofibrils produced from renewable sources and naturally occurring halloysite nanoclay could be used as complementary reinforcing agents. 

In the second group related to Montmorillonite-composites, an interesting article, authored by Choi et al. [5], reported the synthesis of a chitosan–montmorillonite nano-composite material grafted with acrylic acid based on its function in a case study analysis. Fuzzy optimization was used for a multi-criteria decision analysis to determine the best desirable swelling capacity (YQ) of the material synthesis at its lowest possible variable cost. A multi-objective fuzzy optimization showed an innovative approach to determine a solution for the best condition in the material synthesis. Therefore, this approach proved to be a practical method for examining the best possible compromise solution based on the desired function to adequately synthesize a material. Moreover, the incorporation of the criteria of the variable cost in terms of material usage and the cumulative uncertainty of the response successfully ensued essential compromise results in the decision-making process. The development of a sacrificial bond provided unique inspiration for the design of advanced elastomers with excellent mechanical properties, but it was still a big challenge to construct a homogeneous polar sacrificial network in a nonpolar elastomer. In this view, Liu et al. [6] proposed a novel strategy to engineer a multi-ionic network into a covalently cross-linked 1,2-polybutadiene (1,2-PB) facilitated by in situ intercalated organic montmorillonite (OMMT) without phase separation. Overall, their work showed the design of a uniform and strong sacrificial network in the nano-clay/elastomer nano-composite with outstanding mechanical performances under both static and dynamic conditions. Future work will be devoted to further improving the ionic crosslinking density and constructing a stronger sacrificial network to prepare shape memory or self-recovery materials and studying the dynamics of ionic crosslinking. In another article montmorillonites (MMT) were modified by intercalating polyethylene oxide (PEO) macromolecules between the interlayer spaces in an MMT-water suspension system [7]. Shu et al. chose MMT/PEO 80/20 composite as the support platform for immobilization of Pd species in preparing novel heterogeneous catalysts. Their results confirmed that Pd nanoparticles were confined within the interlayer space of MMT and/or dispersed well on the outer surface of MMT. This work offers an alternative approach to the preparation of Pd heterogeneous catalysts with fairly good performances, and heterogeneous catalysts with fairly good performances, and could have broad prospects in both experimental and industrial applications. The effect of doubly functionalized montmorillonite (MMT) on the structure, morphology, thermal, and tribological characteristics of the resulting polystyrene (PS) nano-composites were investigated by Yu et al. [8]. The modification of the MMT was performed using a cationic surfactant and an anionic surfactant or a silane-coupling agent to increase the compatibility with PS matrix. The nanocomposites prepared by a cationic surfactant and a silane-coupling agent exhibited the best thermal stability and tribological performance, providing significant guidance for the future synthesis and application of the PS/OMMT nanocomposites in the oil and gas drilling engineering field to improve drilling fluid lubrication. 

The last part of Special Issue is composed of different papers, which can be collected in a common category namely “*Hybrid Clay Nano-Composites*”. Zhu et al. [9] have prepared Polyimide@graphene oxide (PI@GO) composites by way of a simple solution blending method. The nanoscale hardness and Young’s modulus of the composites were measured using nano-indentation through atomic force microscopy (AFM). They showed that relatively low GO content could remarkably improve the nanoscale mechanical properties of PI and they demonstrated that 2D nano-materials could improve the self-healing performance of polymer composites. In another paper Jiang et al. [10] conjugated hyaluronic acid (HA)—a natural polysaccharide that can specifically bind to CD44 receptors, onto laponite^®^ (LAP) nano-disks for the encapsulation and targeted delivery of the anti-cancer drug doxorubicin (DOX) to CD44-overexpressed cancer cells. Their results demonstrate that the HA-modified LAP nano-disks with high drug loading efficiency, pH-sensitive drug release properties and CD44 targetability might be an efficient nano-platform for cancer chemotherapy. Another interesting work dealing with the adsorption of Atrazine (ATZ) from aqueous solutions using nanocomposite materials, synthesized with two different types of organo-modified clays was written by Jorge A. Ramírez-Gómez et al. [11]. The structural, morphological, and textural characteristics of clays, copolymers, and nano-composites were determined through different analytical and instrumental techniques. They finally demonstrated that the synthesized nano-composites with higher molar fractions of 4VP obtained the highest removal percentages of ATZ. 

The article written by Alexandros K. Nikolaidis et al. [12] covers an interesting area of the application of clay nano-composites: dental materials. It focuses on the reinforcement of dental nano-composite resins with diverse organomodified montmorillonite (OMMT) nanofillers. The aim of this work was to monitor whether the presence of functional groups in the chemical structure of the nanoclay organic modifier may virtually influence the physicochemical and/or the mechanical attitude of the dental resin nano-composites. An enhancement of the flexural modulus was observed, mainly by using clay nanoparticles decorated with methacrylated groups, along with a decrease in the flexural strength at a high filler loading. This work can provide novel information about chemical interaction phenomena between nano-fillers and the organic matrix towards the improvement of dental restorative materials. In the following contribution, Elisabetta Finocchio’s group [13] modified a montmorillonite clay with three different aliphatic polyamines and deeply investigated the interaction mechanisms between clay and amines by different experimental techniques among them X-ray powder diffraction (XRD), thermal analysis measurements (DTG), Fourier Transform Infrared Spectroscopy (FT-IR). Their experimental results showed that the amount of amines efficiently immobilized in the solid phase could be enhanced by increasing the initial concentration of polyamines in the clay modification process, envisaging that polyamine-based organo-clays are promising materials for their proposed application in environmental remediation. Layered silicates are suitable for use as fillers in nano-composites based on a large aspect ratio, easy availability, and chemical resistance. Sericite is distinguished for its higher aspect ratio, higher resilience, and ultraviolet shielding and absorption. In this view, Liang et al. [14] studied the stability of the sericite intercalated by CTAB by changing different washing solvents, different temperatures, ultrasonic cleaning, and different solution conditions. Sericite/polymer nano-composites were produced with the stable intercalated sericite, and demonstrated excellent properties compared with pure epoxy resin. Altogether these results have suggested that stable intercalated sericite is a precondition for good adhesion between the sericite and epoxy resin, which gives rise to good nano-composite mechanical properties. In the contribution of Liu et al. [15] an attapulgite (ATP)/polypyrrole (PPy) nano-composite was developed employing the in situ polymerization method to produce the hierarchical cell texture for the PS foam based on the supercritical CO_2_ foaming. The results showed that the nano-composite could act as an efficient CO_2_ capturer enabling the random release of it during the foaming process. Therefore, the in situ polymerized ATP/PPy nano-composite makes a supercritical CO_2_ foaming desired candidate to replace the widely used fluorocarbons and chlorofluorocarbons as PS blowing agents. Finally, Bugnicourt et al. [16] investigated the effect of various preparation methods on different production scales (pilot- and semi-industrial scale) on the barrier performance and morphological properties of the applied nano-composites. A nano-enhanced composition was converted into a so-called “ready-to-use” formulation by means of a solid-state pre-dispersion process using ball-milling. The preparation of a coating formulation using the ready-to-use granules and its up-scaling for roll-to-roll converting of a pilot- and semi-industrial scale was also successfully implemented. Transmission electron microscopy, scanning electron microscopy, as well as oxygen permeability measurements have been employed to characterize the effects of both the production at various scales and ultrasound treatment on the morphology and barrier performance of the nano-composites. Authors concluded that the solid state pre-dispersion of the nano-platelets during the production of the ready-to-use formulation was the predominant process determining the ultimate degree of nanoparticle orientation and dispersion state.
